# HLA-A and HLA-B genes are involved in the pathogenesis of IBS

**DOI:** 10.1097/MD.0000000000033135

**Published:** 2023-03-03

**Authors:** Huiping Liang, Li Li, Lan Huang, Tingting Lu, Qi Luo, Yanning Mao, Huaying Liu

**Affiliations:** a Department of Medicine, Guangxi Medical College, Nanning, China; b Dean’s Office of Guangxi Medical College, Nanning, China; c Department of Medical Technology, Guangxi Medical College, Nanning, China; d The First People’s Hospital of Nanning, Nanning, China.

**Keywords:** HLA-A, HLA-B, irritable bowel syndrome, protective gene, susceptibility gene

## Abstract

Irritable bowel syndrome (IBS) is the most common functional gastrointestinal disorder. The pathogenesis of IBS has not yet been fully elucidated, and the relationship between human leukocyte antigen (HLA) class I molecules and IBS is not clear. The present case-control study investigated the correlation between HLA-A and HLA-B genes and IBS. Peripheral blood samples were collected from 102 IBS patients and 108 healthy volunteers at Nanning First People’s Hospital. DNA was extracted using a routine procedure, and HLA-A and HLA-B gene polymorphisms were identified by polymerase chain reaction with sequence-specific primers to determine the genotype and distribution frequency of HLA-A and HLA-B in IBS patients and healthy controls. Susceptibility and protective genes for IBS were identified using univariate and multivariate analyses. The frequency of HLA-A11 gene expression in the IBS group was significantly higher than that in the healthy control group, while the frequencies of HLA-A24, 26, and 33 gene expression were significantly higher in the healthy control group than in the IBS group (all *P* < .05). The frequencies of HLA-B56 and 75 (15) gene expression in the IBS group were significantly higher than those in the healthy control group, while the frequencies of HLA-B46 and 48 gene expression were significantly higher in the healthy control group than in the IBS group (all *P* < .05). Genes that may be related to the prevalence of IBS were included in the multivariate logistic regression, and the results suggested that the HLA-B75 (15) gene is a susceptibility gene for IBS (*P* = .031, odds ratio [OR] = 2.625, 95% confidence interval [CI]: 1.093–6.302), while the HLA-A24 (*P* = .003, OR = 0.308, 95% CI: 0.142–0.666), A26 (*P* = .009, OR = 0.162, 95% CI: 0.042–0.629), A33 (*P* = .012, OR = 0.173, 95% CI: 0.044–0.679), and B48 (*P* = .008, OR = 0.051, 95% CI: 0.006–0.459) genes are protective genes for IBS.

## 1. Introduction

Irritable bowel syndrome (IBS), the most common functional gastrointestinal disorder, is characterized by recurrent abdominal pain accompanied by changes in defecation frequency and/or stool characteristics.^[1]^ According to a large transnational study conducted by the Rome Foundation, the overall global prevalence of IBS is 3.8% and 10.1% according to the Rome IV criteria and Rome III criteria, respectively.^[2]^ The prevalence of IBS in China varies by region and population. In the reported literature, the incidence rate varies from 5.9% to 12.7% in China.^[3]^ IBS can seriously affect the ability of patients to work and study and can negatively impact their quality of life and mental health, with an important economic impact on the health care system.^[4]^ The pathogenesis of IBS has not yet been fully elucidated, and IBS has traditionally been considered to result from a combination of factors. Previous studies have confirmed that genetic polymorphisms may play a role in the development and progression of IBS by regulating factors such as the entero-cerebral axis, gastrointestinal motility, inflammatory activity, and immune status.^[5,6]^ Therefore, a large amount of research has been carried out to identify genes related to IBS. Scholars have previously explored the association between HLA class II genes and IBS. Although the reported conclusions are not completely consistent, these studies confirmed that HLA-DQ genes are associated with IBS.^[7–9]^ However, There has been no research report on the influence of HLA class I molecules on IBS, such as HLA-A and HLA-B genes.

The human leukocyte antigen (HLA) system was the first genetic system confirmed to be clearly associated with many diseases.^[7]^ HLA class I molecules are more widely distributed than HLA class II molecules, and their primary physiological function is antigen presentation to CD8^+^ T cells, which account for approximately 80% of the characteristic cells (intraepithelial lymphocytes [IELs]) of the intestinal mucosal immune system distributed between epithelial cells of the small and large intestines. We speculate that HLA-A and HLA-B genes, which are belonged to HLA class I molecules may affect the pathogenesis of IBS by affecting the intestinal immune status or participating in a mechanism linked to other aspects of IBS pathogenesis. To verify the hypothesis, we selected IBS patients and healthy controls from China to participate in this study, detected polymorphisms in the HLA-A and HLA-B alleles by polymerase chain reaction (PCR) with sequence-specific primers, and identified susceptibility genes and protective genes related to IBS in China, providing new insight into the mechanism of IBS.

## 2. Materials and methods

### 2.1. Study participants

From January 2020 to June 2022, IBS patients and healthy volunteers from China were selected from Nanning First People’s Hospital. The inclusion criteria for the IBS group were as follows: met the Rome IV diagnostic criteria for IBS; had not recently taken psychotropic drugs or drugs affecting gastrointestinal function; and all the subjects have undergone colonoscopy to confirm that there are no organic intestinal lesions or other causes that may lead to clinical manifestations similar to IBS, such as inflammatory bowel disease, have been excluded. The healthy control group included healthy physical examinees without a family history of autoimmune diseases or genetic diseases. Written informed consent for the use of clinical records was granted by each patient, as required by the Institutional Review Board of the hospital and college, in accordance with the ethical guidelines stated in the Declaration of Helsinki (1964).

### 2.2. Diagnostic criteria and exclusion criteria for IBS

Diagnostic criteria and exclusion criteria for IBS were based on IBS Rome IV criteria: recurrent abdominal pain at least 1 day/wk (onset within the last 3 months) combined with two or more of symptoms related to defecation, change in defecation frequency, and change in stool characteristics. The above symptoms must have appeared 6 months before diagnosis, and the findings in the last 3 months were required to meet the above criteria. The exclusion criteria were as follows: hypothyroidism, hyperthyroidism, chronic pancreatitis or other diseases that could lead to changes in stool frequency and characteristics and peptic ulcer, abdominal surgery, progressive liver disease, kidney disease, cancer, and other serious diseases.

### 2.3. Specimen collection and processing

Three milliliters of fasting venous blood was obtained from all subjects in the morning. After anticoagulation with ethylenediaminetetraacetic acid, samples were aliquoted at 500 μL/tube and immediately placed at −80 °C for storage.

### 2.4. DNA sample preparation

DNA was extracted from 500 μL of frozen blood using a whole blood genomic DNA extraction kit (Tianjin Xiupeng Biotechnology Development Co., Ltd., Xiqing District, Tianjin, China) according to the instructions. DNA concentration and purity were detected by an ultramicro UV spectrophotometer (Thermo NanoDrop 2000, Thermo Scientific, Wilmington, DE), and samples with a concentration of 12.5 to 100 ng/μL and an A260/A280 ratio between 1.6 and 1.9 were used for PCR analysis.

### 2.5. HLA-A and HLA-B genotyping

HLA-A and HLA-B genotyping was performed by PCR-sequence-specific primers. An HLA genotyping kit from Tianjin Xiupeng was used for PCR according to the instructions. After PCR, 7 μL of each product was separated by electrophoresis in 1.5% agarose at 150 V for 15 minutes.

### 2.6. Observation and interpretation of the results

**Figure 1.**
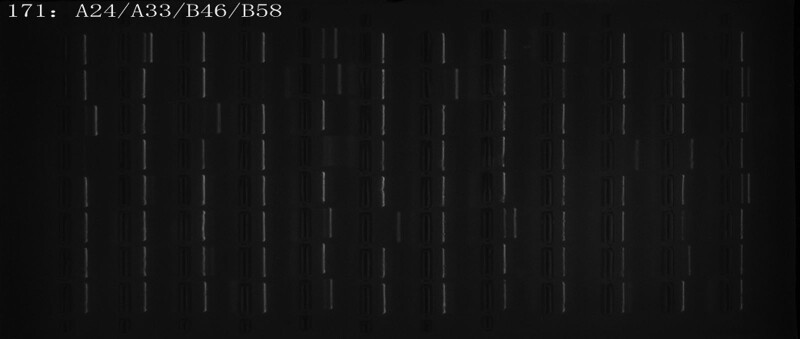
PCR electrophoresis of HLA-A24/33 heterozygotes and HLA-B46/58 heterozygotes. HLA = human leukocyte antigen, PCR = polymerase chain reaction.

**Figure 2.**
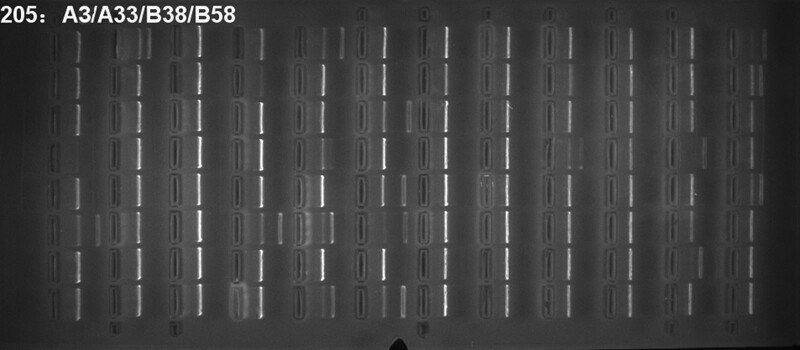
PCR electrophoresis of HLA-A11 homozygotes and HLA-B75(15) homozygotes. HLA = human leukocyte antigen, PCR = polymerase chain reaction.

The genotyping results were imaged using a UV gel imaging system and interpreted according to the result typing table in the kit instructions (Figs. 1 and 2).

### 2.7. Statistical analysis

All data were statistically analyzed using SPSS 22.0 software (IBM Corporation, Armonk, NY). Qualitative (count) data are presented as the rate (%), Pearson χ^2^ test was used to compare 2 groups, and logistic regression was used for the multivariate analysis of IBS-related genes. *P* < .05 was considered to indicate statistical significance.

## 3. Results

### 3.1. Study population and baseline characteristics

A total of 102 IBS patients were enrolled in this study, including 48 (47.06%) with IBS mainly characterized by diarrhea, 13 (12.75%) with IBS mainly characterized by constipation, 25 (24.51%) with IBS with mixed bowel habits, and 16 (15.69%) with IBS of an uncertain type. There were 45 (44.12%) males and 57 (53.88%) females with an average age of 43.94 ± 10.24 years in the IBS group. A total of 108 healthy controls were enrolled, including 47 (43.52%) males and 61 (56.48%) females with an average age of 45.30 ± 12.10 years.

### 3.2. Comparison of HLA-A gene frequencies between IBS patients and healthy controls

Eight HLA-A alleles were detected in both groups, including HLA-A2, 3, 11, 24, 26, 30, 31, and 33. The frequency distribution of HLA-A11 gene expression in the IBS group was significantly higher than that in the healthy control group (57.8% vs 39.8%, *P* = .009), while the frequencies of HLA-A24, 26, and 33 gene expression were significantly higher in the healthy control group than in the IBS group (38.9% vs 19.6%, *P* = .002; 11.1% vs 2.9%, *P* = .022; and 11.1% vs 2.9%, *P* = .022). These data are presented in Table 1.

**Table 1:** The gene frequency of the HLA-A alleles in the patient group and the control group (n %).

HLA gene	IBS group (N = 102)		Control group (N = 108)		χ^2^	*P*	OR	95% CI
	Total	Proportion (%)	Total	Proportion (%)				
A2	62	60.8	59	54.6	0.814	.367	1.287	0.743–2.229
A3	9	8.8	4	3.7	2.368	.124	2.516	0.750–8.442
A11	59	57.8	43	39.8	6.825	.009	2.074	1.196–3.596
A24	20	19.6	42	38.9	9.372	.002	0.383	0.205–0.715
A26	3	2.9	12	11.1	5.279	.022	0.242	0.066–0.886
A30	8	7.8	4	3.7	1.668	.196	2.213	0.645–7.587
A31	2	2.0	5	4.6	0.497	.489	0.412	0.078–2.173
A33	3	2.9	12	11.1	5.279	.022	0.242	0.066–0.886

CI = confidence interval, HLA = human leukocyte antigen, IBS = irritable bowel syndrome, OR = odds ratio.

### 3.3. Comparison of gene frequencies of HLA-B alleles in IBS patients and healthy controls

A total of 19 HLA-B alleles were detected in each group, including HLA-B8, 13, 27, 35, 39, 44, 46, 48, 51, 54, 55, 56, 58, 60, 61, 62, 71 (70), 75 (15), and 76. The HLA-B56 and 75 (15) gene expression frequencies in the IBS group were significantly higher than those in the healthy control group (5.9% vs 0.0%, *P* = .032; and 23.5% vs 11.1%, *P* = .017), while the HLA-B46 and 48 gene expression frequencies were significantly higher in the healthy control group than in the IBS group (51.9% vs 35.3%, *P* = .016; 8.3% vs 1.0%, *P* = .030). These data are presented in Table 2.

**Table 2:** The gene frequency of the HLA-B alleles in the patient group and the control group (n %).

HLA gene	IBS group (N = 102)		Control group (N = 108)		χ^2^	*P*	OR	95% CI
	Total	Proportion (%)	Total	Proportion (%)				
B8	0	0.0	4	3.7	2.124	.145	1.038	1.001–1.078
B13	30	29.4	20	18.5	3.431	.064	1.833	0.961–3.497
B27	1	1.0	4	3.7	0.707	.400	0.257	0.028–2.343
B35	5	4.9	1	0.9	1.727	.189	5.515	0.633–48.043
B39	3	2.9	5	4.6	0.077	.781	0.624	0.145–2.682
B44	3	2.9	4	3.7	0.000	1.000	0.788	0.172–3.610
B46	36	35.3	56	51.9	5.842	.016	0.506	0.291–0.882
B48	1	1.0	9	8.3	4.737	.030	0.109	0.014–0.876
B51	15	14.7	8	7.4	2.865	.091	2.155	0.872–5.327
B54	0	0.0	4	3.7	2.124	.145	1.038	1.001–1.078
B55	9	8.8	8	7.4	0.141	.707	1.210	0.448–3.266
B56	6	5.9	0	0.0	4.592	.032	0.941	0.897–0.988
B58	9	8.8	16	14.8	1.795	.180	0.556	0.234–1.323
B60	21	20.6	19	17.6	0.305	.581	1.214	0.609–2.421
B61	6	5.9	4	3.7	0.174	.677	1.625	0.445–5.934
B62	4	3.9	2	1.9	0.236	.627	2.163	0.388–12.074
B71 (70)	2	2.9	0	0.0	1.472	.225	0.971	0.938–1.004
B75 (15)	24	23.5	12	11.1	5.695	.017	2.462	1.157–5.235
B76	1	1.0	3	2.8	0.200	.655	0.347	0.035–3.387

CI = confidence interval, HLA = human leukocyte antigen, IBS = irritable bowel syndrome, OR = odds ratio.

### 3.4. Logistic regression of IBS-related HLA-A and HLA-B genes

Potential IBS-related genes were included in the multivariate regression analysis. Logistic regression showed that the HLA-B75 (15) gene may be a susceptibility gene for IBS (*P* = .031, odds ratio [OR] = 2.625, 95% confidence interval [CI]: 1.093–6.302), while the HLA-A24 (*P* = .003, OR = 0.308, 95% CI: 0.142–0.666), A26 (*P* = .009, OR = 0.162, 95% CI: 0.042–0.629), A33 (*P* = .012, OR = 0.173, 95% CI: 0.044–0.679), and B48 (*P* = .008, OR = 0.051, 95% CI: 0.006–0.459) genes were identified as protective genes for IBS. These data are shown in Table 3.

**Table 3:** Logistic regression of IBS-related HLA-A and HLA-B genes.

HLA gene	*B* value	Walds	*P*	OR	95% CI
HLA-A11	0.054	0.023	.879	1.056	0.525–2.123
HLA-A24	−1.179	8.958	.003	0.308	0.142–0.666
HLA-A26	−1.821	6.905	.009	0.162	0.042–0.629
HLA-A33	−1.752	6.324	.012	0.173	0.044–0.679
HLA-B46	−0.577	3.246	.072	0.561	0.300–1.052
HLA-B48	−2.979	7.037	.008	0.051	0.006–0.459
HLA-B56	20.907	0.000	.999	1.202E9	0.000
HLA-B75 (15)	0.965	4.662	.031	2.625	1.093–6.302

CI = confidence interval, HLA = human leukocyte antigen, IBS = irritable bowel syndrome, OR = odds ratio.

## 4. Discussion

HLA class I molecules have important immunobiological functions and participate in various immune responses. On 1 hand, HLA class I molecules are directly involved in the processing and presentation of endogenous antigens (viral antigens, intracellular bacterial antigens, tumor antigens, etc.) by antigen-presenting cells (APCs); on the other hand, when CD8^+^ cytotoxic T lymphocytes specifically recognize antigenic peptides presented by APCs, HLA class I molecules bound to antigenic peptides must be recognized at the same time to be activated, and then, adaptive immune responses can be initiated. Recent studies have found that IBS patients show different degrees of changes in the immune system (activation of the immune system/abnormal immune regulation), including decreased innate immune defence and activation of the adaptive immune system.^[10]^ To investigate the correlation between HLA-A and HLA-B genes and IBS, the present study included 102 IBS patients and 108 healthy controls, and logistic regression analysis showed that the HLA-B75 (15) gene may be a susceptibility gene for IBS (*P* = .031, OR = 2.625, 95% CI: 1.093–6.302), while the HLA-A24 (*P* = .003, OR = 0.308, 95% CI: 0.142–0.666), A26 (*P* = .009, OR = 0.162, 95% CI: 0.042–0.629), A33 (*P* = .012, OR = 0.173, 95% CI: 0.044–0.679), and B48 (*P* = .008, OR = 0.051, 95% CI: 0.006–0.459) genes were identified as protective genes for IBS.

The intestinal mucosal immune system is composed of intestinal epithelial cells, intestinal IELs, lamina propria lymphocytes, intestinal submucosal collecting lymph nodes and immune cells such as dendritic cells and mucosal mast cells. Various APCs induce immune responses dominated by interactions between intestinal epithelial cells and intestinal submucosal collecting lymph nodes upon antigen stimulation. Intestinal IELs are distributed between epithelial cells of the small and large intestinal mucosa, close to the intestinal lumen, and are the first sites of contact with bacteria, viruses, and food antigens in the intestinal mucosal immune system. More than 90% of intestinal IELs are T cells, 80% of which are CD8^+^ T cells. Intestinal IELs are mainly divided into 2 categories, aIELs and bIELs, and mucosal bIELs play a role in protecting against mucosal infection by recognizing antigens through NKG2D receptors. In contrast, mucosal aIELs kill infected epithelial cells by specifically recognizing peptides from viruses or other intracellular pathogens presented by major histocompatibility complex-class I molecules on infected epithelial cells. These aIELs induce infected epithelial cell death by releasing perforin and granzyme and can also induce apoptosis of epithelial cells through the Fas/FasL pathway to achieve an anti-mucosal infection effect; however, excessive immune responses can lead to pathological damage of the intestine and disruption of intestinal barrier function.^[7]^ Many studies have found that IBS patients have increased intestinal mucosal infiltration of inflammatory immune cells, such as mast cells, enterochromaffin cells, T lymphocytes, and neutrophils, and this larger population of inflammatory immune cells can release a variety of bioactive substances to induce systemic and local inflammatory cytokine responses.^[11,12]^ Inflammatory cytokines act on the intestinal nerves and immune system, weaken the intestinal mucosa barrier, and trigger IBS symptoms, indicating that an abnormal intestinal mucosal immune response can directly or indirectly affect the intestinal barrier function of IBS patients.

HLA class I molecules can participate in the intestinal mucosal immune response by presenting various antigenic peptides to intestinal IELs, and accordingly, we speculated that HLA-A and HLA-B genes encoding classical HLA class I molecules may be associated with IBS. After detecting polymorphisms in HLA-A and HLA-B alleles in IBS patients and healthy controls, the gene frequency distributions were compared between these 2 groups, and the genes potentially related to the pathogenesis of IBS were included in the multivariate logistic regression equation. The results suggested that the HLA-B75 (15) gene may be a susceptibility gene for IBS (*P* = .031, OR = 2.625, 95% CI: 1.093–6.302), while the HLA-A24 (*P* = .003, OR = 0.308, 95% CI: 0.142–0.666), A26 (*P* = .009, OR = 0.162, 95% CI: 0.042–0.629), A33 (*P* = .012, OR = 0.173, 95% CI: 0.044–0.679), and B48 (*P* = .008, OR = 0.051, 95% CI: 0.006–0.459) genes were identified as potential protective genes for IBS. These results suggest a definite association between IBS and HLA-A and HLA-B genes. The HLA-B75 (15) gene may be a susceptibility gene for IBS in China, while the HLA-A24, 26, 33 and HLA-B48 genes have a protective effect. The mechanism underlying this association may be that overexpression of the HLA-B75 (15) gene leads to abnormal presentation of endogenous antigens, breaking the immune tolerance status of the intestinal mucosa and inducing an excessive protective immune response, which leads to pathological damage of the intestine, destroys the barrier function of the intestine, and increases the risk of IBS. However, the HLA-A24, 26, and 33 and HLA-B48 genes may protect against the occurrence of IBS because of their weak ability to present the corresponding antigens, which renders the host hyporesponsive or unresponsive to harmless antigens and maintains the healthy intestine in an immune tolerant state. In summary, we believe that HLA-A and HLA-B genes mainly affect the prevalence of IBS by influencing the intestinal immune status and immune response, thus directly or indirectly affecting intestinal barrier function.

Multiple complex and interacting factors are simultaneously involved in the pathogenesis of IBS. A review of the pathogenesis of IBS indicates the involvement of factors such as abnormal gastrointestinal motility, visceral hypersensitivity, brain-gut axis interactions, intestinal flora disorders and metabolic abnormalities, gastrointestinal infection and inflammation, psychophysiological factors and central nervous system disorders, food factors, genetics and gene polymorphisms.^[5]^ Therefore, we inferred that HLA-A and HLA-B genes may be additional factors involved in the pathogenesis of IBS. Visceral hypersensitivity is the core pathogenesis of IBS and plays an important role in the development and progression of this disorder. The development of visceral hypersensitivity in IBS involves a complex cascade encompassing disruption of intestinal barrier function, activation of the intestinal immune system; moreover, neuroendocrine system disorders can cause the activation of downstream cytokines and receptors, evoking signaling cascades that impact the central nervous system.^[13]^ A study by Long et al^[14]^ showed that inhibition of this dysfunction or restoration of the intestinal barrier can correct visceral hypersensitivity in IBS.

Patients with IBS often also exhibit manifestations such as anxiety and depression,^[15]^ and a meta-analysis showed that IBS patients had a 3-fold increased chance of anxiety or depression compared with healthy individuals.^[16]^ Psychological factors and the physiological function of the digestive tract interact with each other through the brain–gut axis to change intestinal motility, improve visceral sensitivity, affect intestinal flora, activate the intestinal mucosal inflammatory response, and affect intestinal epithelial cell function. As a disorder of gut–brain interaction, IBS is closely related to stress. Notably, stress can cause sensitization of pain-related higher centers, spinal pathways, and visceral afferent nerves, prompting increased sensitivity of the intestine to normal stimuli at multiple levels. Chronic stress can increase intestinal mucosal barrier permeability, causing endotoxaemia and intestinal or systemic low-grade inflammation.^[17]^ Both acute and chronic stress can induce or aggravate symptoms in IBS patients, resulting in increased intestinal sensitivity, increased levels of inflammation, and disturbances in the hypothalamic–pituitary–adrenal axis. Studies have confirmed that in response to stress, the number of activated mast cells in the intestinal mucosa lamina propria increases in both healthy people and IBS patients, and the release of active substances surges, resulting in increased intestinal mucosal permeability and flora displacement.^[18]^

Most IBS patients exhibit onset or worsening of symptoms after food intake.^[19]^ The effect of diet on individual physiology and disease depends on genetic and microbiota factors; whereas genetic differences affect individual metabolism and nutrient bioavailability, the structure of the microbiome affects microbiota function, metabolites, and metabolic regulation. After ingested protein is degraded by intestinal enzymes, digested soluble antigens are primarily absorbed through intestinal epithelial cells and presented to T cells by APCs; this selectively activates CD8^+^ T suppressor cells, which leads to specific tolerance or intolerance to food protein and can impact the pathogenesis of IBS. A case–control study showed a significant increase in IELs in the IBS group, further confirming that changes in mucosal immune cells are a feature of IBS.^[20]^ The relative abundance of gut microbiota species in IBS patients differs from that in healthy people, the gut of patients with IBS is mainly characterized by alterations in microbiota diversity, mucosa-associated microbiota species, and microbiota proportions.^[21]^ Compared with healthy people, IBS patients tend to have reduced microbiota diversity.^[22]^ The imbalance in the intestinal flora causes an increase in intestinal mucosal permeability, allowing a large number of pathogenic bacteria and their antigens to cross the intestinal barrier and thus readily enter the blood through the intestinal mucosa; these events cause excessive immunity in the host and increase the levels and activation status of a variety of immune cells and inflammatory factors.^[23]^ Thus, intestinal barrier dysfunction in IBS may be associated with visceral hypersensitivity, psychophysiological factors, food factors, brain–gut axis interactions, intestinal flora disorders and metabolic abnormalities; moreover, these factors may interact with each other to affect the pathogenesis of IBS.

The development of molecular biology techniques is rapidly advancing, and genetic research provides broad prospects for the intervention, treatment and prevention of IBS. However, the genes and potential pathways associated with IBS are still not fully understood; multiple complex factors are involved in the pathogenesis of IBS and interact with each other, and the underlying pathogenesis has not yet been completely elucidated.

## 5. Limitations of the study

However, there are certain limitations to our research. First, as this study was limited to the analysis of HLA-A and HLA-B genes, the interference of confounding factors such as other HLA alleles and non-HLA genes cannot be completely ruled out. Second, the results of this study may be affected by various factors, such as subject selection, sample size, and experimental error. Hence, whether HLA-A and HLA-B genes affect IBS as independent factors and whether there is synergy with other factors need to be determined in future studies.

## 6. Conclusion

This study suggests that genetic susceptibility to IBS may be associated with HLA-A and HLA-B gene polymorphisms. The HLA-B75 (15) gene may be a susceptibility gene for IBS, while the HLA-A24, 26, and 33 and HLA-B48 genes may be protective genes for IBS in China.

## Acknowledgments

We would like to thank Mr. Zhizhong Li who is a professor of statistical for collating and analyzing clinical data.

## Author contributions

**Conceptualization:** Huiping Liang, Li Li, Huaying Liu.

**Data curation:** Huiping Liang, Lan Huang, Tingting Lu.

**Formal analysis:** Qi Luo, Yanning Mao, Huaying Liu.

**Investigation:** Lan Huang, Tingting Lu.

**Methodology:** Huiping Liang, Li Li, Huaying Liu.

**Project administration:** Huiping Liang, Li Li, Lan Huang, Huaying Liu.

**Supervision:** Huiping Liang, Li Li, Huaying Liu.

**Writing – original draft:** Huiping Liang, Li Li, Huaying Liu.

**Writing – review & editing:** Huiping Liang, Huaying Liu.

## References

[1] Camilleri M. Diagnosis and treatment of irritable bowel syndrome: a review. JAMA. 2021;325:865–77. 33651094 10.1001/jama.2020.22532

[2] Sperber AD Bangdiwala SI Drossman DA. Worldwide prevalence and burden of functional gastrointestinal disorders, results of Rome foundation global study. Gastroenterology. 2021;160:99–114.e3. 32294476 10.1053/j.gastro.2020.04.014

[3] Liu YL Liu JS. Irritable bowel syndrome in China: a review on the epidemiology, diagnosis, and management. Chin Med J (Engl). 2021;134:1396–401. 34074848 10.1097/CM9.0000000000001550 PMC8213251

[4] Black CJ Ford AC. Global burden of irritable bowel syndrome: trends, predictions and risk factors. Nat Rev Gastroenterol Hepatol. 2020;17:473–86. 32296140 10.1038/s41575-020-0286-8

[5] Weaver KR Melkus GD Fletcher J. Relevance of sex and subtype in patients with IBS: an exploratory study of gene expression. Biol Res Nurs. 2020;22:13–23. 31833409 10.1177/1099800419889189 PMC7068753

[6] Xiao QY Fang XC Li XQ. Ethnic differences in genetic polymorphism associated with irritable bowel syndrome. World J Gastroenterol. 2020;26:2049–63. 32536774 10.3748/wjg.v26.i17.2049 PMC7267697

[7] Debebe BJ Boelen L Lee JC.; IAVI Protocol C Investigators. Identifying the immune interactions underlying HLA class I disease associations. eLife. 2020;9:e54558. 32238263 10.7554/eLife.54558 PMC7253178

[8] Domżał-Magrowska D Kowalski MK Szcześniak P. The prevalence of celiac disease in patients with irritable bowel syndrome and its subtypes. Prz Gastroenterol. 2016;11:276–81. 28053683 10.5114/pg.2016.57941 PMC5209460

[9] Kårhus LL Thuesen BH Skaaby T. The distribution of HLA DQ2 and DQ8 haplotypes and their association with health indicators in a general Danish population. United Eur Gastroenterol J. 2018;6:866–78. 10.1177/2050640618765506 PMC6047278 30023064

[10] Fan WJ Zhang X Fang XC. The immune factors in the pathogenesis of irritable bowel syndrome. Zhonghua Nei Ke Za Zhi. 2018;57:378–80. 29747300 10.3760/cma.j.issn.0578-1426.2018.05.018

[11] Burns G Carroll G Mathe A. Evidence for local and systemic immune activation in functional dyspepsia and the irritable bowel syndrome: a systematic review. Am J Gastroenterol. 2019;114:429–36. 30839392 10.1038/s41395-018-0377-0

[12] Bashashati M Moossavi S Cremon C. Colonic immune cells in irritable bowel syndrome: a systematic review and meta-analysis. Neurogastroenterol Motil. 2018;30. 10.1111/nmo.13192 28851005

[13] Dai L Zhong LL Ji G. Irritable bowel syndrome and functional constipation management with integrative medicine: a systematic review. World J Clin Cases. 2019;7:3486–504. 31750331 10.12998/wjcc.v7.i21.3486 PMC6854423

[14] Long Y Du L Kim JJ. MLCK-mediated intestinal permeability promotes immune activation and visceral hypersensitivity in PI-IBS mice. Neurogastroenterol Motil. 2018;30:e13348. 29644768 10.1111/nmo.13348

[15] Hu Z Li M Yao L. The level and prevalence of depression and anxiety among patients with different subtypes of irritable bowel syndrome: a network meta-analysis. BMC Gastroenterol. 2021;21:23. 33413140 10.1186/s12876-020-01593-5 PMC7791666

[16] Zamani M Alizadeh-Tabari S Zamani V. Systematic review with meta-analysis: the prevalence of anxiety and depression in patients with irritable bowel syndrome. Aliment Pharmacol Ther. 2019;50:132–43. 31157418 10.1111/apt.15325

[17] de Punder K Pruimboom L. Stress induces endotoxemia and low-grade inflammation by increasing barrier permeability. Front Immunol. 2015;6:223. 26029209 10.3389/fimmu.2015.00223 PMC4432792

[18] Bednarska O Walter SA Casado-Bedmar M. Vasoactive intestinal polypeptide and mast cells regulate increased passage of colonic bacteria in patients with irritable bowel syndrome. Gastroenterology. 2017;153:948–60.e3. 28711627 10.1053/j.gastro.2017.06.051 PMC5623149

[19] Spiller R. Impact of diet on symptoms of the irritable bowel syndrome. Nutrients. 2021;13:575. 33572262 10.3390/nu13020575 PMC7915127

[20] Talley NJ Alexander JL Walker MM. Ileocolonic histopathological and microbial alterations in the irritable bowel syndrome: a nested community case-control study. Clin Transl Gastroenterol. 2020;12:e00296. 33464728 10.14309/ctg.0000000000000296 PMC8345925

[21] Wang L Alammar N Singh R. Gut microbial dysbiosis in the irritable bowel syndrome: a systematic review and meta-analysis of case-control studies. J Acad Nutr Diet. 2020;120:565–86. 31473156 10.1016/j.jand.2019.05.015

[22] Duan R Zhu S Wang B. Alterations of gut microbiota in patients with irritable bowel syndrome based on 16S rRNA-targeted sequencing: a systematic review. Clin Transl Gastroenterol. 2019;10:e00012. 30829919 10.14309/ctg.0000000000000012 PMC6407812

[23] Hanning N Edwinson AL Ceuleers H. Intestinal barrier dysfunction in irritable bowel syndrome: a systematic review. Therap Adv Gastroenterol. 2021;14:1756284821993586. 10.1177/1756284821993586 PMC7925957 33717210

